# Frequency of sharp wound debridement in the management of diabetes-related foot ulcers: exploring current practice

**DOI:** 10.1186/s13047-021-00489-1

**Published:** 2021-08-12

**Authors:** Vanessa L Nube, Jennifer A Alison, Stephen M Twigg

**Affiliations:** 1 Department of Podiatry, Royal Prince Alfred Hospital, Sydney, Missenden Rd, Camperdown, NSW 2050 Australia; 2 Faculty of Medicine and Health, Sydney Medical School (Central), The University of Sydney, Camperdown, Australia; 3 Faculty of Medicine and Health, Sydney School of Health Sciences, The University of Sydney, Camperdown, Australia; 4 Allied Health, Sydney Local Health District, Camperdown, Australia; 5 Department of Endocrinology, Royal Prince Alfred Hospital, Sydney, Camperdown, Australia

## Abstract

**Background:**

Conservative sharp wound debridement (CSWD) is fundamental to wound bed preparation. Evidence-based practice guidelines strongly recommend frequent CSWD of diabetes-related foot ulcers (DFU) based on expert opinion and observational studies which suggest that more frequent debridement is associated with better healing outcomes.

**Aim:**

To document current practice with regards to CSWD of DFU and whether this is performed at every visit, how often and what factors determine debridement frequency.

**Method:**

Survey data were collected and managed using REDCap electronic data tools, a secure, web-based application. The survey was distributed through podiatry managers and relevant clinical networks between October 2017 and February 2018.

**Results:**

One hundred clinicians opened the survey and seventy-five surveys were completed by *n* = 53 NSW Health (Australia) employed podiatrists (representing 41% of all NSW Health podiatrists), 11 privately practicing podiatrists, and 11 nurses. Most (*n* = 47) worked in metropolitan areas versus regional/remote (*n* = 28). CSWD was the most frequently used debridement method, performed at every visit by most (84%) of podiatrists. Callus, slough and infection presence were the top 3 most important determinants of frequency, with staff time (a limiting factor) ranking 4th. Regional/remote podiatrists practiced less frequent debridement compared with those in metropolitan areas (debridement every 2 weeks or less = 71% regional podiatrists versus 45% metropolitan podiatrists) (*p* = 0.024).

**Conclusion and clinical implications:**

CSWD was the predominant form of debridement used with debridement occurring at every treatment visit for most of the clinicians surveyed. Debridement frequency was determined by clinical wound indications and staffing resources, with regional/remote podiatrists providing debridement less often than their metropolitan colleagues.

**Supplementary Information:**

The online version contains supplementary material available at 10.1186/s13047-021-00489-1.

## Introduction

Diabetes-related foot ulcers (DFU) are a highly prevalent, chronic wound type which place considerable burden on the healthcare system, the patient, and often their family. Prompt access to interdisciplinary management is critical to address the aetiological factors and to promote healing. Debridement of the wound to prepare the wound bed for endogenous healing is a fundamental component of care and several potential methods of debridement are described including surgical and non-surgical modalities [1, 2].

Current evidence for sharp debridement is based on observational studies using retrospective analyses [3–5] and is rated as weak, due to a lack of prospective studies of the efficacy of debridement [2]. Notwithstanding the lack of prospective randomised studies, conservative sharp wound debridement is a mainstay of treatment provided by podiatrists and some nurses with specialised training within the Australian Health Care System. Sharp debridement of DFU is cited in leading management guidelines from European and Canadian and US organisations and the International Working Group on the Diabetic Foot as standard care [2, 6–9] . It is performed using a scalpel, forceps and curette to achieve rapid and concise removal of non-viable tissue and senescent cells from the base and edge of the wound to facilitate healing. While the evidence is unclear about the extent to which bacterial load is reduced with debridement [10], the current consensus is that serial debridement has a key role in managing infected wounds and biofilm which is associated with most chronic wounds [11, 12]. Hyperkeratosis, a particular feature of diabetes-related foot ulcers associated with loss of sensation and chronic repetitive trauma is also managed with sharp debridement. The removal of hyperkeratosis is associated with a reduction in plantar pressure [13, 14] which is likely to support healing.

Retrospective studies suggest a positive, dose-dependent relationship between the extent and frequency of debridement. Performed serially when there is adequate blood flow for healing, several studies have shown as association between more frequent sharp debridement and improved healing outcomes. This has provided incentive, if not direct evidence, for thorough and frequent, sharp debridement of DFU [3–5, 15].

The model of care for providing treatment of DFU involves clinic visits to assess and manage the wound and factors associated with healing such as offloading, management of infection, diabetes and education of the patient. The provision of CSWD is likely to be strong determinant of visit frequency, for which there is no direct substitute. More frequent treatment visits for the purpose of CSWD places more demand on the clinical workforce which would need to considered in the context of a small and finite workforce. Current clinical practice regarding debridement frequency and factors that influence clinical decision making concerning debridement are not known.

The aim of this survey was to document current debridement practice in the management of diabetes-related foot ulcers by podiatrists, and factors associated with debridement frequency. The objectives of the survey were to determine debridement type and frequency, specifically: whether CSWD is the most frequently used method of debridement; how often DFU are debrided; whether DFU are debrided at every clinic visit; whether there are differences in debridement frequency between the regional and metropolitan areas what factors influence clinicians’ decisions with regards to how often ulcers are debrided. These data were collected while a randomised study of debridement frequency (Clinical trial registration: ACTRN12618000703202) was being undertaken by the authors. The findings of the survey were planned to inform implementation strategies for translation of evidence from the randomised trial into practice.

## Method

The survey was quantitative, cross-sectional and respondents were recruited through relevant networks via an email invitation which provided a link to the online survey. The online survey was developed de novo by the first author (VN). The prototype survey was provided to 10 podiatrists including the site investigators of the aforementioned randomised debridement study who manage DFU from both metropolitan and regional areas, and a podiatry academic. Feedback as to what aspects of the prototype survey could be modified to minimise ambiguity was provided. Respondents indicated which questions should be modified in order to enable a refined survey to adequately address the study aims and this was incorporated into the final version (Additional file 1: Survey Questions). In addition to closed questions and discrete data, survey participants were asked to “Please make any additional comments here regarding the factors that influence your debridement frequency, the enablers or barriers”.

The online survey was developed using REDCap, an electronic data tools and secure, web-based application hosted at the Sydney Local Health District. Survey data were subsequently managed using REDCap [16] An invitation to participate including a URL to the final version of the online survey was distributed between October 2017 and February 2018.

To target New South Wales (NSW) Health podiatrists, the survey invitation was distributed to Podiatry Managers and to individual podiatrists known to be engaged in the care of people with diabetes-related foot complications within High Risk Foot Services via the state-based Community of Practice. Members of the Community of Practice are clinicians with an interest in management of patients with diabetes-related foot complications. While the membership is not exclusively podiatrists, they are the main discipline represented. So as not to exclude private practitioners or nurses involved in debridement, the survey was also distributed to 30 NSW private practice attendees at a Diabetes Foot Education session, and wound care nurses within the lead site were also provided a link which they could disseminate to their peers.

The invitation advised potential participants that they were invited to participate on the basis that they were involved in the management of diabetes-related foot ulcers, that the project was funded by the Ministry of Health to help improve understanding about current debridement practice and that it could be completed anonymously and take approximately 10 min to complete. An email address was provided for additional information.

No identifying information, name practice or institutional details were required for the survey. A URL was provided at the end of the survey in which participants were invited to provide their contact information. This URL was not linked to the survey responses and was to aid the researchers in knowing who had responded. Twenty three participants submitted their details using this URL.

Data were included for analysis if participants responded ‘yes’ to managing non-ischaemic DFU with conservative sharp wound debridement and if they provided responses to the following: number of DFU treated on average each week; how often they debrided individual foot ulcers; whether they debrided at every treatment visit; whether they were located (rural, regional or metropolitan); and their clinical discipline.

Participants were also provided the option to obtain and use a simple audit tool to record information relating the ulcers treated, the frequency of debridement, and healing presence at 12 weeks. Consent to participate in the audit relied on an additional layer of approval from their site Governance and Research office. The protocol, audit tool, questionnaire and participant information sheets were approved by the Sydney Local Health District Human Research and Ethics Committee – Concord Repatriation and General Hospital (LNR 17CRGH112 CH 62/6/2017–076). The information sheet available from the survey link advised that consent to use the respondents’ survey data was enacted when the participant proceeded to complete the survey.

## Results

One hundred participants opened the survey link, seventy five met the inclusion criteria, and 70 completed all questions. Characteristics of the participants are detailed in Table 1.

**Table 1:** Characteristics of participants who opened and those whose responses were included in the analysis

	All participants (who opened survey) ***n*** = 100	Participants included in analyses ***n*** = 75
**Location of service**		
Metropolitan	58 (64.4%)	47 (62.7%)
Rural	24 (26.7%)	22 (29.3%)
Remote	8 (8.9%) N = 10 missing data	6 (8%)
**Health sector**		
Public – Community Health	16 (18%)	11 (14.7%)
Public – Hospital	61 (68.5%)	52 (69.3%)
Private	12 (31.5%)	11 (14.7%)
Other	N = 1 missing data	N = 1 missing data
**Discipline**		
Podiatrist	66 (77.6%)	64 (85.3%)
Nurse	19 (22%) *N* = 15 missing data	11 (14.7%)
**Years of podiatry experience**		
Mean	13.3	13.3
Median	13	13
Range * Podiatrists only	1–45 years	1–45 years
**Number of clients treated with DFU each week**		
< 1 per week	17 (20%)	13 (17.3%)
1–4 per week	15 (17.6%)	12(16%)
> 4 and < 10 per week	17 (20%)	15 (20%)
10 or more per week	36 (42.4%) N = 15 missing data	35 (46.7%)

Most of the respondents were podiatrists, the majority (83%) of whom were employed by NSW Health. The number of NSW Health employed podiatrists who completed the survey (*n* = 53/129) represented 41% of the NSW Health podiatry workforce according to 2018 data provided by the NSW Department of Health, Workforce Branch. For podiatrists employed in the public sector, half reported that more than 60% of their caseload was the management of DFU and 28% reported DFU management was 90% or more of their caseload. Podiatrists employed in the private sector and nurses treated proportionally fewer people with DFU (Table 2).

**Table 2:** Number of diabetes-related foot ulcers treated per week (on Average)

Weekly patient number treated (average)	Public Sector Podiatrists (*n* = 53)	Private Sector Podiatrists (*n* = 11) 10% missing data	Nurses (*n* = 11)
Less than 1	3	7	3
Between 1 and 4	6	2	5
From 5 to 10	12	1	2
More than 10 patients	32	1	2

All respondents performed CSWD in their management of DFU with CSWD being the predominant method of debridement used. Most (73%) reported using other forms of debridement infrequently. The exception was hydrogels which were used at least occasionally by most respondents (Table 3).

**Table 3:** Use of other (non-sharp) methods of wound debridement

	Never		Occasionally		Sometimes		Often		Always	
	Podiatrists NSW Public sector	All	Podiatrists NSW Public sector	All	Podiatrists NSW Public sector	All	Podiatrists NSW Public sector	All	Podiatrists NSW Public sector	All
Hydrogel	3	5	30	36	13	18	3	8	1	2
LFUD	44	60	2	3	4	6	4			
Versajet	49	68	1	1						
Larvae	48	62	2	6				1		

Missing Data Podiatrists NSW = 3

Missing Data Overall = 7

*LFUD* = Low frequency ultrasonic debridement

Versajet = Hydrosurgical debridement therapy

### Frequency of conservative sharp wound debridement

**Fig. 1:**
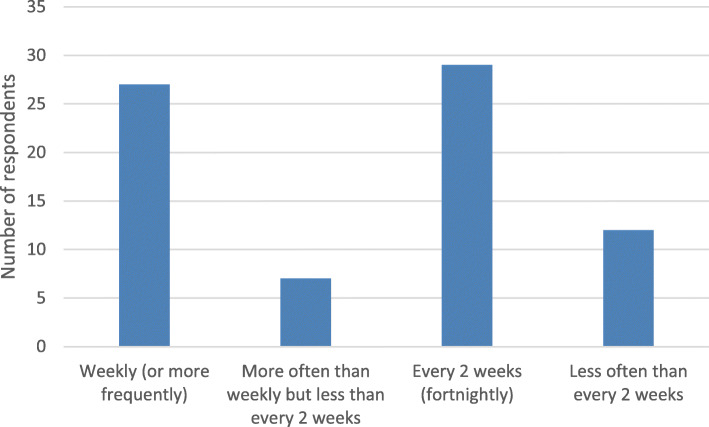
The average frequency of conservative sharp wound debridement of diabetes-related foot ulcers as reported by clinicians

Most respondents performed CSWD at weekly (29%) or fortnightly intervals (39%) with a small number of respondents (7%) debriding their patients’ DFU more frequently than this or at longer intervals of up to 5 weeks (16%) (Fig. 1).

### Debridement at every treatment visit

Most respondents (79%) performed CSWD in the treatment of DFU at every visit if the patient had adequate blood flow. Of the podiatry respondents, 84% performed CSWD at every visit and of the NSW Health employed podiatrists, 92% debrided at every visit. NSW Health employed podiatrists who reported not debriding at every visit (*n* = 4), debrided their patients’ ulcers fortnightly (*n* = 2), weekly (*n* = 1) and one debrided more frequently than once a week. Nurses were less likely to debride at every visit.

### Differences between rural/regional and metropolitan clinicians

Regional/remote clinicians were more likely to debride their patients’ ulcer less often with 71% reporting performing CSWD of their patient’s DRFU every 2 weeks or less often compared with 45% of metropolitan clinicians z = 2.25, *p* = 0.024. Using data for NSW Health employed podiatrists; regional/remote practicing podiatrists practice less frequent debridement with 68% reporting performing SWD of their patients every 2 weeks or less often compared to 35% of metropolitan podiatrists z = 2.35, *p* = 0.019.

### Factors that determined frequency of conservative sharp wound debridement

In reporting the relative importance of different factors to consider when determining debridement frequency, callus was rated as very important by most respondents (97%) followed by slough (76%), infection (59%), lack of clinical staff time (51%), patient non-adherence to attendance (44%), transport access (33%), consultation fee (23%), and transport/parking costs (20%).

**Fig. 2:**
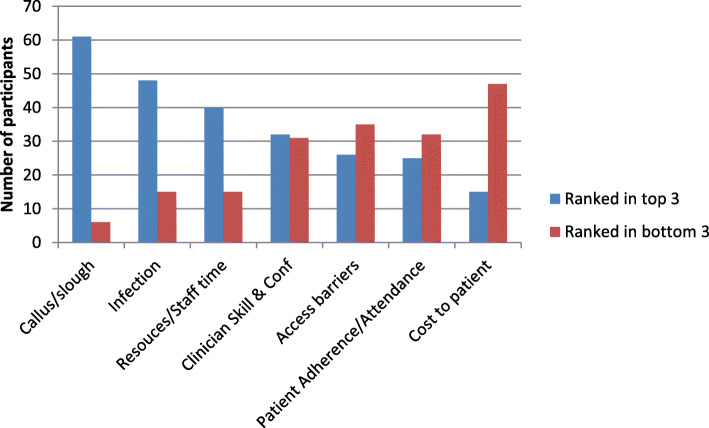
Respondent ranking of importance of the variables influencing debridement frequency. The top 3 rankings and bottom 3 rankings combined for each variable and data ordered from those ranked as most important (blue) to least important (red)

Respondents were also asked to rank (in order of importance), the factors they considered in determining debridement frequency. Potential indications of callus and slough were combined for this question. The presence of callus and slough, infection, and clinical staff time/resources were more frequently ranked in the top three considerations for determining debridement frequency of non-ischaemic DFU (< 10% missing data) (Fig. 2).

## Discussion

Based on NSW Health reported numbers, the respondents represent 41% of NSW Health employed podiatrists, which for a survey process is a high percentage. This high response rate indicates that the survey sample is likely to be representative of the public podiatry workforce in NSW. The number of private podiatry and nurse respondents was low and less likely to be representative of these groups however they were not the main target populations for this study. Notably, NSW Health employed podiatrists represent a small proportion (11%) of the registered podiatrists in NSW and are more likely to be engaged in the care of DFU than their private practice colleagues [17].

Previous surveys have explored clinician practice and have reported on wound debridement. In their online survey of Australian podiatrists designed to determine adherence to evidence-based practice guidelines for managing DFU, Quinton (2015) asked respondents if they performed sharp debridement on non-ischaemic wounds. The median response from public sector employed podiatrists nationally was “always” and their private practice colleagues reported debriding “very often”. The respondents (*n* = 310) included 48 NSW podiatrists (18 public: 30 private). An international survey of clinicians engaged in management of chronic wounds, reported by Swanson (2017) found that 57% of clinicians frequently used sharp debridement, and 46% of Australian clinicians frequently used sharp debridement in the management of biofilm [18]. The respondents (*n* = 2614) represented a broad cross section of clinicians; while not reported, the proportion of podiatrists as a small profession, would be expected to be low.

The results of this study indicate that conservative sharp wound debridement is a mainstay of treatment and the predominant modality used to remove non-viable tissue in the management of DFU. Other modalities were rarely used, with the exception of hydrogel which was used at least occasionally. Due to the frequent debridement performed, it is likely that hydrogels are used as an adjunct to sharp debridement and/or to restore moisture to a dry wound.

CSWD is currently provided at every treatment visit to a NSW Health employed podiatrist for a patient with diabetes-related foot ulceration. While treatment visits are most commonly every 1 or 2 weeks, clinical indications are used to determine frequency, together with consideration of the available clinic resources to provide care. In the case of rural and regional areas, patients are more likely to receive the treatment less often and this is likely to be a consequence of staff resources and potentially distance from the patients’ homes to the clinic. The latter has not been explored directly with patients but relatively few of our respondents rated transport issues as very important in this survey.

## Conclusions

Podiatrists employed within the public health system are providing conservative sharp wound debridement to all patients with non-ischaemic diabetes-related foot ulcers as the predominant modality to remove non-viable tissue to promote healing. The frequency of debridement is virtually synonymous with treatment visit frequency as wounds were debrided at every visit, with frequency determined by clinical indicators but limited in some cases by available resources.

Patients receiving care in rural and regional settings were also provided with this treatment however they were most likely to receive debridement every 2 weeks or less often when compared to those attending metropolitan based services who were more likely to receive weekly debridement. While this data is informative as to current practice, whether such debridement frequency of weekly vs less often impacts ulcer healing in people with diabetes, will require a prospective, randomised trial to address this key clinical question.

## Supplementary Information

**Additional file 1.**

## Data Availability

Please contact author for data requests. We did not request data sharing in our original ethics application however if required, we can seek approval for the dataset to be made available on reasonable request to the author.

## Abbreviations

CSWD: Conservative sharp wound debridement
DFU: Diabetes-related foot ulcer
NSW: New South Wales

## References

[1] Kirshen C, Woo K, Ayello EA, Sibbald RG (2006). Debridement: a vital component of wound bed preparation. Adv Skin Wound Care.

[2] Rayman G, Vas P, Dhatariya K, Driver V, Hartemann A, Londahl M (2020). Guidelines on use of interventions to enhance healing of chronic foot ulcers in diabetes (IWGDF 2019 update). Diabetes Metab Res Rev.

[3] Steed DL, Donohoe D, Webster MW, Lindsley L (1996). Effect of extensive debridement and treatment on the healing of diabetic foot ulcers. Diabetic ulcer study group. J Am Coll Surg.

[4] Cardinal M, Eisenbud DE, Armstrong DG, Zelen C, Driver V, Attinger C, Phillips T, Harding K (2009). Serial surgical debridement: a retrospective study on clinical outcomes in chronic lower extremity wounds. Wound Repair Regen..

[5] Wilcox JR, Carter MJ, Covington S (2013). Frequency of debridements and time to heal: a retrospective cohort study of 312 744 wounds. JAMA Dermatol.

[6] Strohal R (2013). The EWMA document: debridement. J Wound Care.

[7] Botros M, Kuhnke J, Embil J, Goettl K, Morin C, Parsins L (2019). Best practice recommendations for the prevention and management of diabetic foot ulcers.

[8] Hingorani ALG, Henke P, Meissner MH, Loretz L, Zinszner KM (2016). The management of the diabetic foot: a clinical practice guideline by the Society for Vascular Surgery in collaboration with the American podiatric medical association and Society for Vascular Medicine. J Vasc Surg.

[9] Wounds UK (2014). Expert working group. Effective debridement in a changing NHS: a UK consensus.

[10] Kim PJ, Attinger CE, Bigham T, Hagerty R, Platt S, Anghel E, Steinberg JS, Evans KK (2018). Clinic-based debridement of chronic ulcers has minimal impact on bacteria. Wounds..

[11] Wolcott R, Rumbaugh KP, James G, Schultz G, Phillips P, Yang Q (2010). Biofilm maturity studies indicate sharp debridement opens a time- dependent therapeutic window. J Wound Care.

[12] Schultz G, Bjarnsholt T, James GA, Leaper DJ, McBain AJ, Malone M (2017). Consensus guidelines for the identification and treatment of biofilms in chronic nonhealing wounds. Wound Repair Regen.

[13] Young MJ, Cavanagh PR, Thomas G, Johnson MM, Murray H, Boulton AJM (1992). The effect of callus removal on dynamic plantar foot pressures in diabetic patients. Diabet Med.

[14] Pataky Z, Golay AM, Faravel L, Silva J, Makoundou V, Peter-Riesch B (2002). The impact of callosities on the magnitude and duration of plantar pressure in patients with diabetes mellitus: a callus may cause 18,600 kilograms of excess plantar pressure per day. Diabetes Metab.

[15] Saap LJ, Falanga V (2002). Debridement performance index and its correlation with complete closure of diabetic foot ulcers. Wound Repair Regen.

[16] Harris PA, Taylor R, Thielke R, Payne J, Gonzalez N, Conde JG (2009). Research electronic data capture (REDCap) – a metadata-driven methodology and workflow process for providing translational research informatics support. J Biomed Inform.

[17] NSW Ministry of Health Workforce Development and Plannning Branch, NSW Health Workforce Branch (2019). Allied Health Factsheets.

[18] Swanson T, Wolcott RD, Wallis H, Woodmansey E (2017). Understanding biofilm in practice: a global survey of health professionals. J Wound Care.

