# Supplementary material for: Frequency of sharp wound debridement in the management of diabetes-related foot ulcers: exploring current practice
Source: J Foot Ankle Res. 2021 Aug 12;14:52. doi: 10.1186/s13047-021-00489-1 (PMC8359122; doi:10.1186/s13047-021-00489-1)
Supplement: Supplementary file 1 — Additional file 1. [file 13047_2021_489_MOESM1_ESM.pdf]

Page 1

Service type where you provide Conservative Sharp Wound Debridement (CSWD) in management of Diabetes-Related Foot Ulcers (DFU)

Where is your service located?Sydney Metropolitan Area

○
○
○ Regional Area
Outer regional or rural area

(A separate survey will collect more specific information but will not be linked to your responses to this survey.)

In which type of service are your providing treatment for Diabetes Related Foot Ulcers (DFU)?

○ Private Practice
○ Community Health Centre
○ Public Hospital
○ Other

In which type of service are your providing treatment for DFU? __________________________________

Your discipline and experience.

Are you a podiatrist?Yes

○
○ No

How many years have you been practicing as a podiatrist?

____
(If less than one year, enter 1.)

What is your clinical discipline? (For example, Nurse, Vascular consultant, General Practitioner)

____

How many patients with Diabetes-Related FootLess than 1 a Ulceration would you treat in a week (on average)?1- 4 per

○ week (on average)
○
○ week
○
More than 4 and less than 10 a week

More than 10 a week
(Please answer for the number of patients you personally treat on average each week (with DFU))

What proportion of your overall patient caseload is treatment of Diabetes-related Foot Ulcers (DFU)? treat	○ Less than 10% of patients I treat<br>○ Between 10% and 30% of patients I<br>○ More than 30% but less than 60%<br>○ More than 60% but less than 90%<br>○ Most of the patients I treat / 90% or more (Please answer for the service where you are engaged in treating people with DFU or the one in which you see the highest proportion of people with DFU)
Optional:<br>What (if any) additional qualifications do you hold in Wound Care or High Risk Foot Management?	☐ Podiatrist level 3 or above - clinical expert<br>☐ Masters in Wound Management field<br>☐ Credentialed in HRF management<br>☐ Credentialed in advanced wound care<br>☐ Other<br>☐ Not applicable<br>(You may select more than one option.)
What is the title of your qualification?	______________________

Conservative Sharp Wound Debridement (CSWD) in treating Diabetes-Related Foot Ulcers (DFU).

Do you perform Conservative Sharp Wound Debridement (CSWD) in your care of patients with DFU?	○ Yes<br>○ No<br>(It is assumed that ulcers where there is severe ischaemia (such that there is insufficient blood flow for healing) are not sharp debrided.)
What is your reason for not performing CSWD in management of DFU?	○ I use other forms of debridement only<br>○ I never encounter DFU in my clinical practice<br>(If you don't use CSWD in DFU management your survey will end here. Thank you.)
If no, what is the reason that you do not perform CSWD in your management of DFU?	______________________

When treating patients with DFU, do you usually perform CSWD at every visit (in most circumstances) ?	○ at every visit/consultation<br>○ not at every visit<br>(A yes response assumes that you debride every DFU at every visit, most of the time. (excluding ischaemic wounds))
How frequently do you debride each patient's DFU (on average)?	○ More frequently than once a week (multip times a week) ○ Once a week<br>○ More often than once a week but less than every 2 weeks ○ Once every 2 weeks<br>○ Once every 3 weeks<br>○ Once every 4 weeks<br>○ Once every 5 weeks or less often<br>(Consider a few of your most recent patients DFU.)

In your opinion and experience, how important are the following factors in determining how frequently you debride a diabetes-related foot ulcer?

Please rate the degree of importance from Not Important to Very important.
It is assumed that these factors would increase the frequency of debridement.

	Not Important		Sometimes		Very Important
Presence of slough	○	○	○	○	○
Presence of callus	○	○	○	○	○
Presence of infection	○	○	○	○	○

What is the effect of the presence of peripheral arterial disease on your decisions regarding debridement frequency?

- ○ Not a factor
- ○ Debride less frequently
- ○ Debride more frequently
- ○ Debride same frequency but remove less tissue
- ○ Other
- ○ don't know

What is the effect of the presence of PAD on your decisions regarding debridement frequency?

In your opinion and experience, how important are the following factors in determining debridement frequency?

Please rate the degree of importance from Not Important to Very Important.
It is assumed these factors would reduce the frequency of debridement.

	Not Important		Sometimes		Very Important
Lack of access to transport	○	○	○	○	○
Cost of transport and parking	○	○	○	○	○
Cost of consultation fee	○	○	○	○	○
Patient non-adherance to appointment attendance	○	○	○	○	○

To what extent are your clinic resources (staffing/time/financial) a determinant in CSWD frequency in treatment of DFU?
It is assumed that these conditions would reduce frequency of debridement.

Please rate as Not Important to Very important.

	Not Important	Sometimes/some	Very important	what important	Lack of clinical staff time
Other financial constraints	○	○	○	○	○
Lack of staff skill and confidence with debridement	○	○	○	○	○
	○	○	○	○	○

Rank factors that influence debridement frequency.

1 is the MOST influential

7 is the LEAST influential factor

Try to choose each response of 1, 2, 3, 4, 5, 6 & 7 only once.

Presence of callus, slough

____

Staff skill and confidence with debridement

____

Resources (including staff time) of clinical service

____

Costs to patient of attending the clinic (including transport and parking)

____

Barriers to access (mobility and transport)

____

Presence of infection

____

Is conservative sharp wound debridement the only method of debridement you use?

○ Yes
○ No

Patient adherance to appointment attendance

____

Please make any additional comments here regarding the factors that influence your debridement frequency, the enablers or barriers.

Use of other methods of debridement in treating Diabetes-related foot ulcers.

How often do you use these other methods in your clinical practice? Rate from Never to Always.

	Never	Occasionally (< 10% of ulcers)	Sometimes (30-50% of ulcers)	Often (50-90% of ulcers)	Always (>90 %)
Hydrogels	○	○	○	○	○
Ultrasound	○	○	○	○	○
Versjet(TM)	○	○	○	○	○
Larvae	○	○	○	○	○

Clinician resources for professional development

To what extent did your undergraduate education prepare you to provide CSWD inprofession confidence?

- I was highly prepared at entry to the (podiatry) study
- I was somewhat prepared, then treating DFU, safely and with "learned on the job"
- I felt inadequately prepared
- not applicable

What evidence-based practice guidelines (EBG) / resources have you used to inform your debridement practice?

- [ ] Australian EBG Diabetic Foot- NHMRC Endorsed, 2011
- [ ] International working group on diabetic foot (IWGDF)
- [ ] Australian Diabetic Foot Network (ADS) guidelines(2012)
- [ ] Other

What other guidelines or resources have informed your debridement practice?

Part of this Ministry of Health funded project is to deliver resources and share our results to inform clinical practice. Is there anything you would like the project team to consider with regards to this? Please take a moment to write your thoughts or recommendations to the team.
